# Bronchus-blocked ultrasound-guided percutaneous transthoracic needle biopsy (BUS-PTNB) for intubated patients with severe lung diseases

**DOI:** 10.1186/s13054-021-03782-4

**Published:** 2021-10-14

**Authors:** Yuean Zhao, Faming Jiang, He Yu, Ye Wang, Zhen Wang, Peng Sun, Zhong Ni, Weiya Wang, Lili Jiang, Junping Fan, Lanlan Zhang, Charles A. Powell, Zongan Liang

**Affiliations:** 1 Department of Respiratory and Critical Care Medicine, West China Hospital, Sichuan University, No. 37, Guo Xue Alley, Chengdu, 610041 Sichuan China; 2 Department of Pathology, West China Hospital, Sichuan University, Chengdu, China; 3 Department of Pulmonary Medicine, Peking Union Medical College Hospital, Peking Union Medical College and Chinese Academy of Medical Sciences, Beijing, China; 4 Division of Pulmonary, Critical Care and Sleep Medicine, Icahn School of Medicine at Mount Sinai, New York, NY USA

**Keywords:** Needle biopsy, Endobronchial blocker, Intubation, Sonography, Bronchoscopy

## Abstract

**Background:**

Examinations based on lung tissue specimen can play a significant role in the diagnosis for critically ill and intubated patients with lung infiltration. However, severe complications including tension pneumothorax and intrabronchial hemorrhage limit the application of needle biopsy.

**Methods:**

A refined needle biopsy technique, named bronchus-blocked ultrasound-guided percutaneous transthoracic needle biopsy (BUS-PTNB), was performed on four intubated patients between August 2020 and April 2021. BUS-PTNB was done at bedside, following an EPUBNOW (evaluation, preparation, ultrasound location, bronchus blocking, needle biopsy, observation, and withdrawal of blocker) workflow. Parameters including procedure feasibility, sample acquisition, perioperative conditions, and complications were observed. Tissue specimens were sent to pathological examinations and microbial tests.

**Results:**

Adequate specimens were successfully obtained from four patients. Diagnosis and treatment were correspondingly refined based on pathological and microbial tests. Intrabronchial hemorrhage occurred in patient 1 but was stopped by endobronchial blocker. Mild pneumothorax happened in patient 4 due to little air leakage, and closed thoracic drainage was placed. During the procedure, peripheral capillary hemoglobin oxygen saturation (SPO_2_), blood pressure, and heart rate of patient 4 fluctuated but recovered quickly. Vital signs were stable for patient 1–3.

**Conclusions:**

BUS-PTNB provides a promising, practical and feasible method in acquiring tissue specimen for critically ill patients under intratracheal intubation. It may facilitate the pathological diagnosis or other tissue-based tests for intubated patients and improve clinical outcomes.

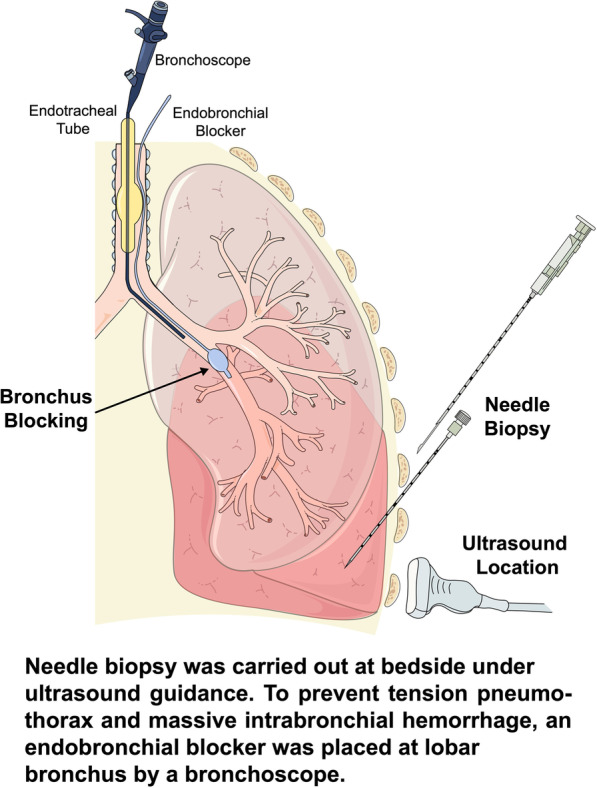

## Introduction

Despite strong life support that modern intensive care may provide, rapid identification of etiology for patient- and cause-specific treatment is vital for patients in a critical condition, especially those on invasive ventilation via endotracheal intubation [1–3]. Pathological studies, usually carried out by lung biopsy, are considered as the gold standard for the diagnosis of many underlying diseases, such as malignancy and mycosis. It is also helpful to diagnose interstitial lung disease, viral infection, tuberculosis and so on. Recently, microbial next-generation sequencing (mNGS) is used for the pathogenic testing in infectious lung diseases. A tissue-based mNGS result may be more representative and accurate than that based on sputum, lower airway suctions, and bronchoalveolar lavage fluid (BALF) in revealing the pathogens of intended lesions [4, 5]. However, biopsy is challenging and risky. Open lung biopsy for confirming a pathological diagnosis of a severe patient is relatively traumatic, difficult to implement, and risk-taking [6, 7]. Cryobiopsy performed on severe patients has been reported to contain excessive bronchial tissues and the peripheral regions seem inaccessible to a bronchoscope [8].Transbronchial lung biopsy (TBLB) to obtain lung tissue is also difficult in severe cases, due to high risks of bleeding and tension pneumothorax under positive pressure ventilation. Besides, the specimen sometimes is likely too sparse for testing [9, 10]. Percutaneous transthoracic needle biopsy (PTNB) is known for obtaining satisfactory peripheral lung tissues, but the risks for pneumothorax and massive intrabronchial bleeding are even higher [11].

To reduce the risks of hemoptysis in TBLB or cryobiopsy for a patient without severe conditions, we often preset an endobronchial blocker, a balloon carried into the bronchus by a tube for blocking, in the targeted lobar bronchus. When a patient starts to bleed out, the balloon can be immediately inflated to block the airway [12]. Thus, we hypothesized that endobronchial blocker could as well be employed for preventing hemoptysis and pneumothorax in critically ill patients undergoing PTNB. When the target lobar bronchus is blocked with an endobronchial blocker, air leakage is theoretically preventable and intrabronchial bleeding may be instantly stopped. Combined with bronchus blockage and ultrasound guidance, PTNB can be carried out to obtain adequate tissue samples with a higher success rate and greater safety at bedside.

We describe this refined technique in the present study, named bronchus-blocked ultrasound-guided percutaneous transthoracic needle biopsy (BUS-PTNB), which we performed on four intubated critically ill patients with lung infiltration. We obtained satisfactory tissue specimens for pathological and pathogenic studies, with acceptable complications during and after procedure.

## Methods

The study was approved by the Institutional Ethics Board of West China Hospital, Sichuan University (approval number: 2021-595). Informed consents were signed with the patients or their family.

### Subjects of study

BUS-PTNBs were performed on intubated patients when a pathological examination of the lung lesions was needed. Patients with a platelet count < 50 × 10^9^/L, an international normalized ratio > 1.5 or a fibrinogen < 1 g/L were excluded [13]. The procedure feasibility, acquisition of satisfactory samples, perioperative conditions, and complications were observed. Samples were sent to pathological studies and tissue-based mNGS. Nine patients were initially evaluated. BUS-PTNB was performed in four patients between 28 August 2020 and 22 April 2021. The procedure was canceled in the other five patients due to new clinical findings strongly indicating a confirmed diagnosis (*n* = 2), disease rapid deterioration (*n* = 2), and families’ disapproval (*n* = 1).

Patients’ vital signs and SPO_2_ were monitored, and all the procedural parameters were also documented. Post-procedure sonography and repeated radiographs within 24 hours were performed to identify potential pneumothorax. Intra- and post-operative bronchoscopy was done for detecting intrabronchial hemorrhage. While performing PTNB, we defined hemorrhage occurring proximal to the blocker as proximal intrabronchial hemorrhage (PIH), which was considered as a biopsy-related complication of clinical significance. Another type of bleeding, which was named as distal intrabronchial hemorrhage (DIH), occurred in the bronchus distal to the blocker. For the blocker can effectively prevent the bleeding from flooding into main airway, DIH is reckoned safe and acceptable for BUS-PTNB.

### Preprocedural conditions of patients

Patient 1, a 48-year-old male, was admitted to the medical intensive care unit (MICU) for rapidly exacerbating dyspnea. He had recurrent irritant coughing accompanied by dyspnea for six months. Chest computerized tomography (CT) showed diffuse patchy ground glass opacity with local interstitial changes over bilateral lung fields. He was diagnosed with severe pneumonia and was given empiric anti-bacterial and anti-fungal regiments. Without significant improvement, BUS-PTNB was performed on the 4th hospitalization day for pathological and pathogenic tests.

Patient 2, a 53-year-old male, was admitted to the MICU for worsening dyspnea. He was diagnosed with severe pneumonia in another hospital and showed no response to antibiotics. Chest CT showed diffuse interstitial changes and consolidation in the lower lobes. BUS-PTNB was performed on the 7th hospitalization day for tissue-based microbial tests.

Patient 3 was a 67-year-old male, who was admitted to the MICU for an intermittent fever and worsening dyspnea. He was previously diagnosed with bronchiectasis and had deep vein thrombosis during hospitalization. mNGS detected nontuberculous mycobacteria (NTM) in his sputum, but CT failed to yield any typical findings. BUS-PTNB was performed on the 4th day since disease worsened.

Patient 4 was a 71-year-old male. He was admitted for worsening dyspnea and rapidly progressing respiratory failure. CT showed diffuse infiltration and honeycomb pattern in the lower lobes. Blood mNGS indicated tuberculosis (TB), which was disagreeable with the severity of his condition. To confirm the diagnosis, BUS-PTNB was performed on the 3rd day since entering MICU (Table 1).

**Table 1:** Patients's preprocedural clinical information

Patient	Age (yrs)	Gender	Underlying disease	Microbial tests		Initial diagnosis	VT (ml)/PEEP(cmH_2_O)	PaO_2_ to FiO_2_ ratio	APACHE II
				Sample	Results				
Patient 1	48	M	–	BALF	PCP	Pneumonia	420/10	185.2 (92.6/50)	17
Patient 2	53	M	VTE	sputum	Candida glabra	Pneumonia	400/10	96 (76.8/80)	11
				sputum	Acinetobacter Baumannii				
Patient 3	67	M	Bronchiectasis	sputum	Aspergillus fumigatus	NTM	480/5	210.5 (84.2/40)	35
				sputum	Klebsiella pneumoniae				
Patient 4	71	M	–	sputum	Candida albicans	TB	450/10	131 (91.7/70)	7

*VT* tidal volume, *PEEP* positive end expiratory pressure, *PaO*_*2*_ oxygen tension, *FiO*_*2*_ fraction of inspiration O_2_, *APACHE II* acute physiology and chronic health evaluation II, *BALF* bronchoalveolar lavage fluid, *PCP* pneumocystis carinii pneumonia, *VTE* venous thrombus embolism, *NTM* nontuberculous mycobacteria, *TB* tuberculosis

### BUS-PTNB procedure

**Fig. 1:**
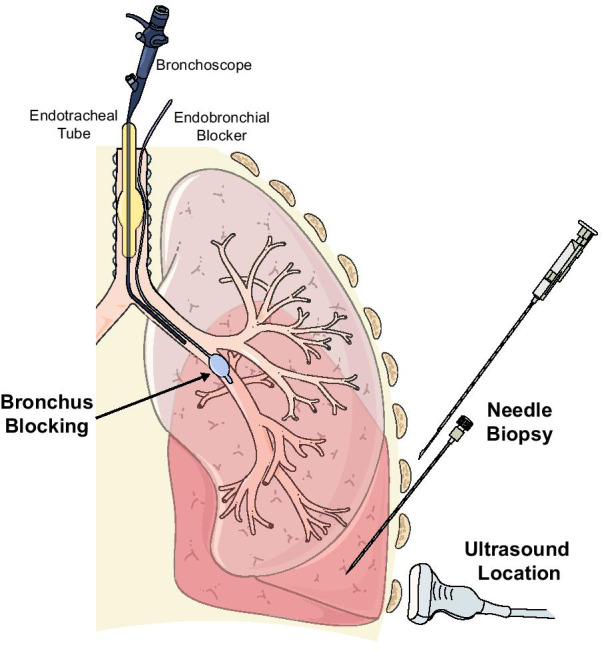
Key Steps of BUS-PTNB. Needle biopsy was carried out at bedside under ultrasound guidance. To prevent severe complications, mainly tension pneumothorax and massive intrabronchial hemorrhage, an endobronchial blocker was placed at lobar bronchus by a bronchoscope while sampling

The procedure of BUS-PTNB was carried out following an EPUBNOW (evaluation, preparation, ultrasound location, bronchus blocking, needle biopsy, observation, and withdrawal of blocker) workflow (Fig. 1).

*Evaluation* Patient’s pre-procedural conditions, such as lesion location on chest CT, respiratory and oxygenation status, were carefully evaluated by three experienced pulmonologists. The intended lobe and patient positioning were decided based on preprocedural chest CT.

*Preparation* Pre-procedural preparations included administration of analgesics and sedatives and sputum aspiration. Fraction of inspiration O_2_ (FiO_2_) was adjusted to 100%, and other ventilator parameters remained unchanged. Patients were properly positioned for BUS-PTNB (supine for anterior chest wall puncture, lateral position for lateral or posterior chest wall puncture). Instruments including bronchoscope, endobronchial blocker, axial guide needle, and cutting needle were carefully checked and prepared. Precautionary first-aid measures were also available at the bedside, which included chest tube, self-inflating resuscitator, epinephrine, and hemostatics.

*Ultrasound location* The precise location for needle insertion was determined under ultrasound guidance. Since lesion was close to the chest wall, high-frequency probe was chosen. Due to its high resolution, the distribution of lung tissue and the surrounding area was clearly shown.

*Bronchus Blocking* Positive end expiratory pressure (PEEP) was adjusted to 5 cm H_2_O and then the endotracheal tube balloon was loosened. The endobronchial blocker (C-AEBS-9.0-78-SPH-AS, Cook Incorporated, USA) was placed into the target lobar bronchus under the guidance of bronchoscope through the para-tubular pathway. Then the bronchoscope was withdrawn and re-entered through the endotracheal tube. After adjusting the blocker position, the blocker’s and the tube’s balloons were both inflated, and PEEP was adjusted back to the baseline level. Air leakage was monitored by the V-T curve on ventilator and presence of respiratory sound at anterior chest. The patient’s SPO_2_ was observed for 5 min, and the ventilator parameters, such as tidal volume, PEEP and FiO_2_, were optimized accordingly. If there was no obvious decrease of SPO_2_, needle biopsy was then conducted. Otherwise, the procedure should be ceased.

*Needle biopsy* Sonography was performed again to verify the puncture site and measure insertion depth. After that, a 17G coaxial guide needle (Max-Core C1816A, BD, USA) was inserted with an appropriate depth after disinfection, with or without ultrasound real-time supervision. Then an 18G cutting needle (Max-Core MC1816, BD, USA) was used to obtain two to four lung tissue specimens, which were sent for pathological examinations and NGS testing.

*Observation* After puncture, the endobronchial blocker was kept in site in case there was any bleeding. The patient was maintained in a supine position and observed for 20 min. Patient’s vital signs and ventilation parameters were continuously monitored throughout the procedure. Sonography was performed to see if there was any pneumothorax. Another bronchoscopy was performed to check if bleeding recurred when loosening the blocker balloon, especially in patients administered with low molecular weight heparin (LMWH) or anti-platelet agents. If active bleeding is detected when loosening the balloon, the endobronchial blocker should be reinflated and kept in place.

*Withdrawal of blocker* Under the supervision of bronchoscope, the endobronchial blocker was withdrawn if active hemorrhage or pneumothorax was not noted. Follow-up bronchoscopy should be scheduled post-procedurally within 24 h to check the bronchi and remove the blood clot if necessary.

### Pathological examination and NGS testing

For pathological examination, specimens were collected and fixed in 10% neutralized formalin solution. Hematoxylin and eosin staining (H&E staining) and immunohistochemistry were performed using standard techniques. Acid-fast staining, TB quantitative polymerase chain reaction (TB-qPCR) and Epstein–Barr encoding region (EBER) in situ hybridization were used when necessary.

For NGS testing, lung tissue specimen was freshly collected. Kits including TIANamp Micro DNA Kit (DP316, TIANGEN BIOTECH, Beijing, China) and Qubit dsDNA HS Assay Kit (Q32854, Thermo Fisher Scientific Inc, USA) were used. After conducting DNA library and data quality control following manufacturer’s protocol, comparison was made between the remaining data and microbial databases to obtain the sequence number matching a certain pathogen, according to which the possible pathogen is finally determined.

## Results

### BUS-PTNB procedures

BUS-PTNB was successfully performed on the four patients and no procedure was attempted but failed or was aborted. The operating team included two physicians (a critical care pulmonologist with 17 years of clinical experience, one of two interventional pulmonologists with 17 or 11 years of clinical experience), two respiratory therapists (one for holding the bronchoscope and the other for operating the endobronchial blocker), and two nurses. All biopsy specimens were sufficient for pathological studies and NGS testing.

**Fig. 2:**
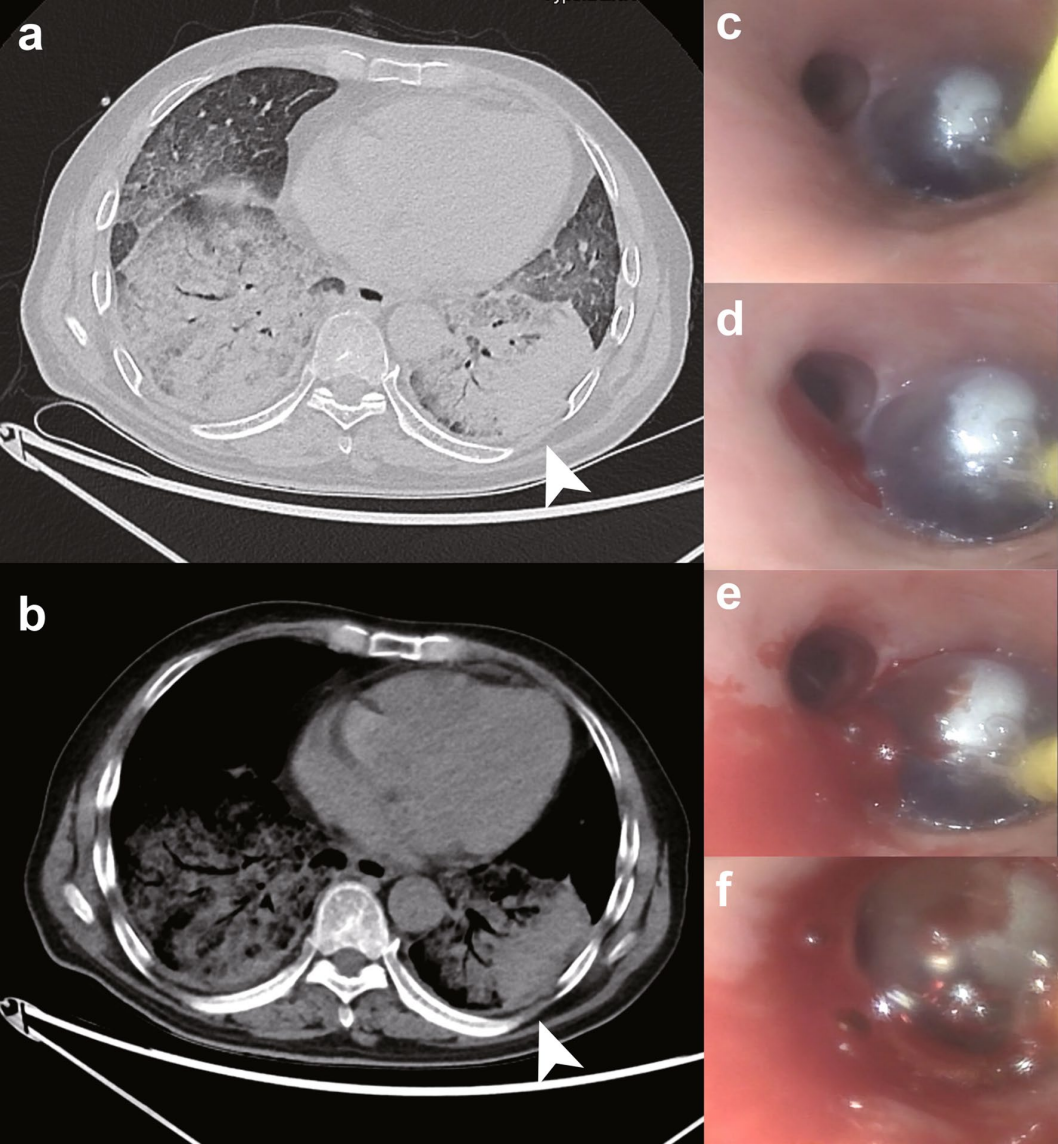
Biopsy site and vision of bronchial blocking under bronchoscopy for patient 1. **a**, **b** Chest CT of patient 1 showed diffuse patchy ground glass opacity with local interstitial changes over bilateral lung fields. Arrowheads indicate the site of puncture. **c** Bronchoscopy showing the endobronchial blocker was initially placed in the common basal segmental bronchus. **d**, **e** Then bleeding occurred in the dorsal segmental bronchus. **f** The endobronchial blocker was withdrawn backwards to inferior lobar bronchus to stop bleeding

For patient 1, needle biopsy was made in the left lower lobe from the backside (Fig. 2a, b). The posterior basal segment was chosen for puncture. He was placed in a right decubitus position. The endobronchial blocker was originally pre-placed in the common basal segmental bronchus (Fig. 2c). However, PIH occurred from the dorsal segmental bronchus during cutting needle sampling (Fig. 2d, e). Thus, the operator pulled the blocker backwards to cover the dorsal segment and no further bleeding occurred (Fig. 2f). The endobronchial blocker was withdrawn 6 h later. Post-procedural bronchoscopy showed no further hemorrhage.

**Fig. 3:**
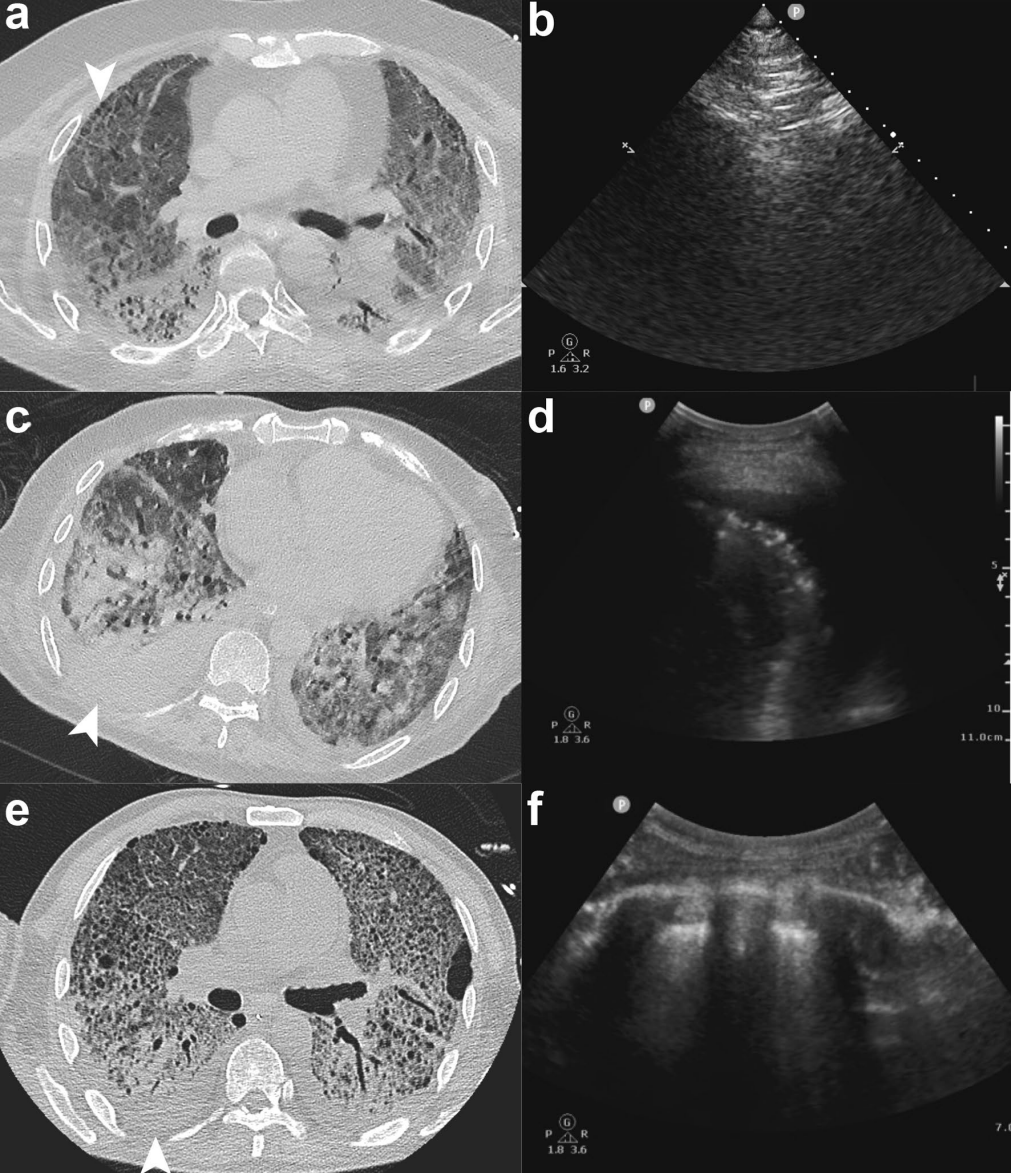
Biopsy sites for patient 2, 3 and 4. **a**, **b** Needle biopsy site was carefully chosen based on CT evaluation before procedure and sonography during the procedure. Right anterior segment was chosen for biopsy in patient 2. **c**, **d** And right posterior basal segment was chosen in patient 3. **e** For patient 4, right dorsal segment was chosen for puncture. **f** After closed thoracic drainage, sonography showed no air left in thoracic cavity

For patient 2, right anterior segment was chosen for BUS-PTNB (Fig. 3a, b), therefore patient was placed on supine position and right upper lobar bronchus was pre-blocked. LMWH was administrated for the management of deep vein thrombosis. Still, no PIH was found. The blocker was withdrawn 5 h later, during which blood clot distal to the blocker was found, indicating little DIH during BUS-PTNB, which was successfully blocked by the blocker.

For patient 3, BUS-PTNB was undertaken in the right posterior basal segment (Fig. 3c, d), and we pre-blocked right lower lobar bronchus. The blocker was withdrawn 5 h later, same as patient 2. However, 36 h later, fever occurred on patient 3 with the highest temperature being 38.1 °C.

For patient 4, right dorsal segment was chosen for needle biopsy (Fig. 3e). Pneumothorax occurred on insertion of coaxial guide needle, probably due to the blocker balloon’s failure to fully block the bronchus and thus resulting in mild air leakage. The balloon was further inflated before the remaining procedure proceeded. Closed thoracic drainage was placed after procedure. Repeated sonography showed no residual air (Fig. 3f). The blocker was withdrawn 2 h later.

### Periprocedural statuses of patients

Table 2 shows the patients’ vital signs. Vital signs of patients 1, 2, and 3 were stable during the procedure. SPO_2_, blood pressure, and heart rate fluctuated in patient 4, who had pneumothorax. His vital signs returned to normal after timely interventions. Patients 1 and 3 had fever after BUS-PTNB. 24 h after BUS-PTNB, fever occurred on patient 1 with the highest temperature of 39.5 ℃, while patient 3 had fever 36 h later.

**Table 2:** Perioperative vital signs and clinical conditions

Patient	SpO_2_	Vital Signs	Operative Parameters				Complications		
	Before/during/after (%)	T(°C)/BP(mmHg)/HR(bpm)/RR(bpm)	Blocker placement duration (h)	Operation duration (min)	Patient position	Lobe/segment	PNX	PIH	DIH
Patient 1	98/100/97	39.5/126/85/85/14	6	30	Right	LL/S10	−	+	−
Patient 2	98/93/92	36.0/117/89/89/26	5	90	Supine	RU/S3	−	−	+
Patient 3	99/94/99	38.1/119/69/111/28	5	60	Left	RL/S10	−	−	−
Patient 4	99/81/96	36.2/113/74/107/28	2	150	Left	RL/S6	+	−	−

*T* temperature, *BP* blood pressure, *HR* heart rate, *RR* respiratory rate, *LL* left lower, *RU* right upper, *RL* right lower, *S10* posterior basal segment, *S3* anterior segment, *S6* dorsal segment, *PNX* pneumothorax, *PIH* proximal intrabronchial hemorrhage, *DIH* distal intrabronchial hemorrhage

Duration of procedure ranged from 30 to 150 min, defined as the time from positioning the patient to withdrawing the bronchoscope. The procedure for patient 4 was longer due to management of mild pneumothorax. The blockers were withdrawn after 5–6 h for all patients except patient 4, whose blocker was removed after 2 h when closed thoracic drainage for pneumothorax was performed.

### Pathological studies and microbial tests

**Fig. 4:**
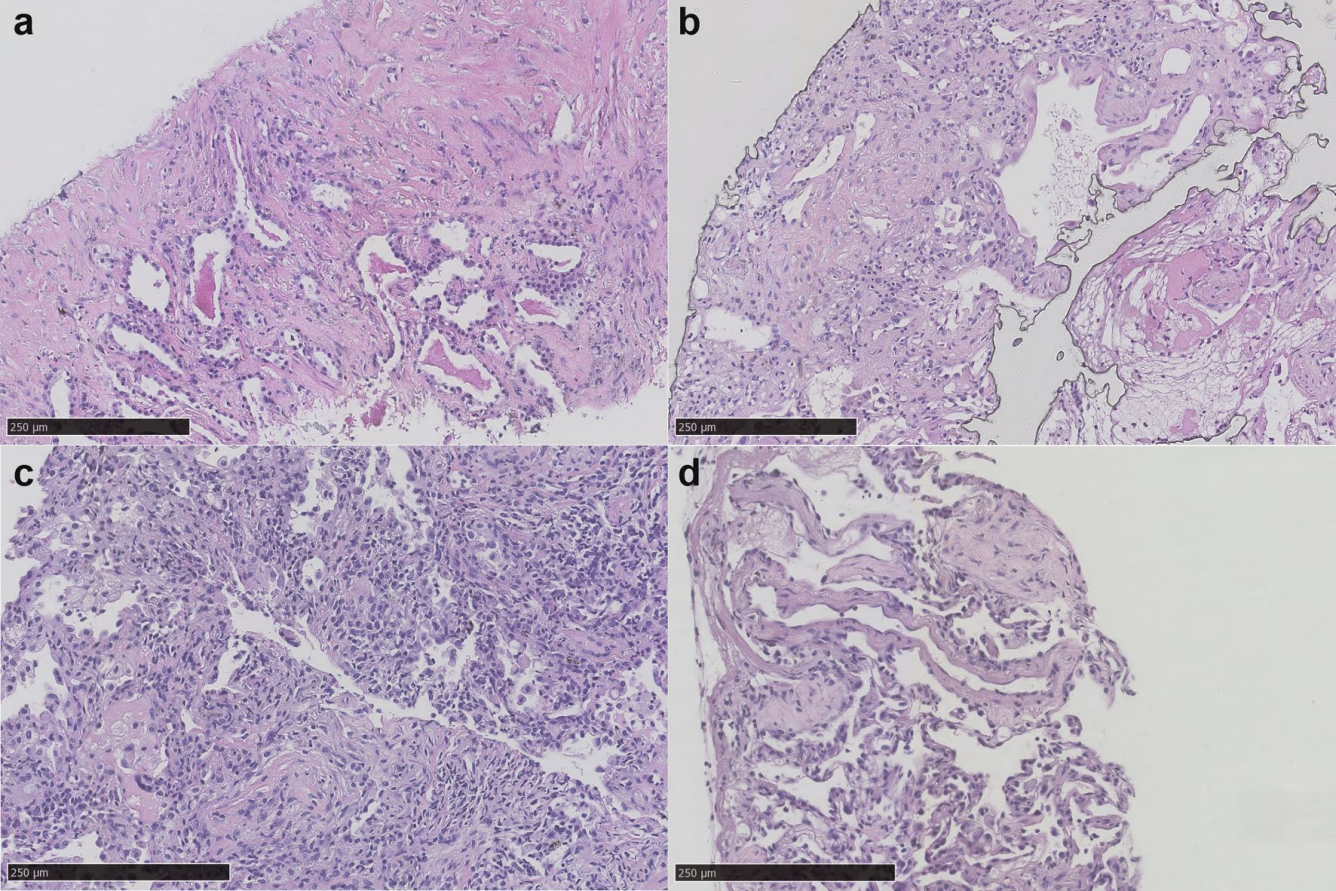
Hematoxylin and eosin staining of acquired tissue specimen. **a** H&E staining of patient 1 showed non-specific inflammation accompanied with interstitial fibrosis with lymphocyte and plasma cell infiltration. Mild alveolar epithelial hyperplasia can be observed and red staining materials aggregated in alveolar lumen with dispersed tissue cells. **b** H&E staining of patient 2 showed alveolar hyaline membrane formation with large nuclei hyperchromatic cells in the alveolar space. **c** H&E staining of patient 3 showed alveolar epithelial and interstitial fibrous tissue hyperplasia, inflammatory exudate, foam cell aggregation and inflammatory cell infiltration in alveolar cavity. **d** H&E staining of patient 4 showed focal chronic inflammation with fibrous hyperplasia

H&E staining showed non-specific inflammation in patient 1 with interstitial fibrosis accompanied with lymphocyte and plasma cell infiltration (Fig. 4a). mNGS revealed cytomegalovirus (CMV) (267 reads), Epstein–Barr virus (EBV) (3 reads) and pneumocystis (9 reads). As a result, antiviral regiment and sulfamethoxazole were additionally given to cover CMV and pneumocystis carinii pneumonia (PCP).

For patient 2, though previously mNGS showed human herpesvirus type I (HSV-1) in BALF and acinetobactor baumannii in blood, they were both negative in tissue mNGS nevertheless. Also taking his clinical features not indicating sepsis into account, we considered that acinetobactor baumannii found in blood was probably due to contamination. H&E staining showed alveolar hyaline membrane formation with large nuclei hyperchromatic cells in the alveolar space, indicating acute lung injury secondary to viral infection (Fig. 4b). Combining pathological findings and NGS results, a viral infection was thus suspected, for which acyclovir was thus administered.

Patient 3 also showed non-specific inflammation on H&E staining (Fig. 4c) and no granuloma was discovered. Results of acid-fast staining and TB-qPCR were negative in lung tissue. Tissue mNGS detected CMV whereas previous BALF mNGS test showed aspergillus, NTM and CMV. Considering all the results above, no therapeutic change was made for this patient.

In patient 4, H&E staining showed focal chronic inflammation with fibrous hyperplasia (Fig. 4d). EBV was positive in both tissue and blood mNGS (3 and 18 reads, respectively). EBER1/2 in situ hybridization result was negative. Acid fast staining was negative and TB-qPCR was positive. TB infection was considered to be a more likely diagnosis. Acinetobacter baumannii was detected by mNGS in BALF but not in tissue or blood, which was considered to be a contaminant due to absent clinical manifestations of sepsis. Anti-TB therapy and glucocorticoids were given to the patient.

## Discussion

In the current study, we report a novel application of needle biopsy, BUS-PTNB, to intubated patients with severe lung diseases. In this new integrate methodology, bronchus blockage is adopted for preventing pneumothorax and intrabronchial hemorrhage during PTNB. The technique allows for acquisition of tissue specimens for pathological studies and other diagnostic tests.

For severely ill patients, the diagnosis is mostly based on clinical manifestations, radiological findings, and examinations of blood and lower respiratory tract specimens. Pathological study is usually lacking for most intubated patients due to the high risks of pneumothorax and intrabronchial bleeding during biopsy. Tension pneumothorax is a serious complication of lung biopsy on positive pressure ventilation [14, 15]. Another life-threatening complication is intrabronchial hemorrhage, which is particularly difficult to manage in intubated patients. Biopsy safety is a main concern of carrying out an early and confirmative pathological diagnosis in ICU.

To circumvent pneumothorax and intrabronchial hemorrhage, a major innovation of BUS-PTNB is introducing the endobronchial blocker into puncture procedure. To prevent air leakage from the airway into the thoracic cavity, we used an endobronchial blocker to block the target bronchus. Meanwhile, the endobronchial blocker can also prevent intrabronchial bleeding. As patient 1 presented, needle biopsy can cause bleeding into the airway, whereas the endobronchial blocker can limit the hemorrhage to the distal end of the balloon, preventing blood from spreading into other lobes and avoiding suffocation. Because DIH is controllable, we considered it safe and acceptable. But sometimes hemorrhage occurred proximal to the blocker due to its inability to cover the target bronchus or its source being other adjacent bronchus. The so-called PIH needs to be managed immediately by adjusting the balloon to the appropriate position and suctioning the blood in time.

The blocker was delivered to target bronchus by a bronchoscope alongside the endotracheal tube. There was insufficient space for advancing both through the endotracheal tube. It was not feasible to use a smaller bronchoscope for this procedure because of the impact of increased friction induced by two instruments on operations, and insufficient capacity for suctioning in case of intrabronchial bleeding. So, we placed the blocker aside the endotracheal tube instead. For withdrawing the blocker, the whole process must be done under the supervision of bronchoscope. If active bleeding is detected when loosening the balloon, the endobronchial blocker should be reinflated and kept in place, especially in patients administered with LMWH or anti-platelet agents.

It’s worth noting that the balloons should be placed in the lobar bronchus instead of the segmental bronchus. Because pulmonary segments in a lobe are interlinked, hemorrhage spreading to adjacent lung segments cannot be prevented. As in the first patient, puncture at the posterior basal segment correspondingly resulted in dorsal segment hemorrhage. On the other hand, obstruction of the lobar bronchus may result in a higher probability of ventilation-perfusion ratio imbalance that may exacerbate respiratory failure. Therefore, for patient selection in the current study, we deem that patient’s SPO_2_ with bronchus blockage should be monitored for 5 min before biopsy. If the SPO_2_ level drops below 90% in bronchus-blocked state, the procedure should be ceased and re-evaluated. In our study, we didn’t see apparent drop of SPO_2_ after bronchus blockage in four patients, partly because we didn’t adjust the inspiratory pressure or PEEP to a particularly low level during the procedures. There’s no need to make such adjustment, for the blockage of bronchus could prevent the positive pressure transducing to the distal end.

Lung disease in the setting of severe respiratory failure usually involves multiple lobes, or is diffusely distributed. Lateral or posterior basal segments of lower lobes are most commonly involved, followed by anterior parts of upper lobe and middle lobe. Regional puncture site in these areas can be easily determined by surface landmarks based on latest preprocedural CT, so as to identify the lobar bronchus to be blocked. Puncture for posterior segments is slightly more risky due to potential injury of the oblique fissure, but lesions involving posterior segments of upper lung solely seldom result in respiratory failure. For a bedside needle biopsy, sonography before inserting the needle can confirm the puncture site, reveal the change of lung lesions, detect the pleural effusion and pneumothorax and observe diaphragm, large vessels and other organs. It can improve the accuracy and safety of bedside needle biopsy, but it cannot precisely determine the specific lobe in which the lesion is located. For the patient was put on pure oxygen ventilation for 2 min, the residual oxygen in the targeted lobe may be quickly absorbed after bronchus blocking. However, no typical sign of atelectasis was observed, probably because the included patients had diffused or extensive lung infiltration involving almost the whole lobe.

Pathological studies for intubated patients can be widely popularized if biopsy safety is promised. Not only can a tissue specimen acquired by BUS-PTNB enable histological studies, immunohistochemistry and special staining, it is also useful for further studies under electron microscope. Besides, tissue specimens are more representative and convincing to clarify the underlying pathogeny, for microbial test from sputum, BALF and blood may be contaminated by colonized or background microbes. Early performance of BUS-PTNB may provide more confirmative information and lead to earlier and more accurate interventions.

However, BUS-PTNB still needs further improvement. First, the endobronchial blocker had to be inserted alongside the endotracheal tube, which increases the complexity of the procedure. We will continue to explore using thinner endobronchial blockers and smaller size bronchoscopes in the subsequent studies. Second, if SPO_2_ is falling below 90% after bronchus blocking despite adjustment of ventilator parameters, the patient might not be suitable for BUS-PTNB due to higher risk of hypoxemia, which would exclude a certain proportion of intended patients. Presently, bronchoscopy must be done repeatedly for surveillance or removing clot. As more experience is acquired, the frequency of bronchoscopy can probably be reduced.

Our study has limitations. Only four patients were included in the current study, all of whom were under endotracheal intubation for a long time and multiple line treatment had been already given. More patients throughout the course of disease, especially at early stage should be included in future studies.

## Conclusions

BUS-PTNB is a promising technique for intubated critically ill patients with lung diseases for its safety and increased amount of specimen collected. It may improve patient’s window of opportunity for etiology-specific treatment and survival by expediting the diagnostic process to yield a confirmed diagnosis.

## Data Availability

The datasets used and/or analyzed during the current study are available from the corresponding author on reasonable request.

## Abbreviations

BALF: Bronchoalveolar lavage fluid
BUS-PTNB: Bronchus-blocked ultrasound-guided percutaneous transthoracic needle biopsy
CMV: Cytomegalovirus
CT: Computerized tomography
DIH: Distal intrabronchial hemorrhage
EBER: Epstein–Barr encoding region
EBV: Epstein–Barr virus
EPUBNOW: Evaluation, preparation, ultrasound location, bronchus blocking, needle biopsy, observation, and withdrawal of blocker
FiO_2_: Fraction of inspiration O_2_
H&E staining: Hematoxylin and eosin staining
HSV-1: Human herpesvirus type I
LMWH: Low molecular weight heparin
MICU: Medical intensive care unit
mNGS: Microbial NGS
NGS: Next-generation sequencing
NTM: Nontuberculous mycobacteria
PCP: Pneumocystis carinii pneumonia
PTNB: Percutaneous transthoracic needle biopsy
PIH: Proximal intrabronchial hemorrhage
SPO_2_: Peripheral capillary hemoglobin oxygen saturation
TB: Tuberculosis
TBLB: Transbronchial lung biopsy
TB-qPCR: TB quantitative polymerase chain reaction
